# Draft Genome Sequence of *Komagataeibacter europaeus* CECT 8546, a Cellulose-Producing Strain of Vinegar Elaborated by the Traditional Method

**DOI:** 10.1128/genomeA.01231-15

**Published:** 2015-10-22

**Authors:** María José Valera, Anja Poehlein, María Jesús Torija, Frederike S. Haack, Rolf Daniel, Wolfgang R. Streit, Estibaliz Mateo, Albert Mas

**Affiliations:** a Biotecnologia Enológica, Departamento de Bioquímica i Biotecnologia, Facultat d’Enologia, Universitat Rovira i Virgili, Tarragona, Spain; b Laboratorium für Genomanalyse, Institut für Mikrobiologie und Genetik, Georg-August-Universität Göttingen, Göttingen, Germany; c Abteilung für Mikrobiologie und Biotechnologie, Biozentrum Klein Flottbek, Universität Hamburg, Hamburg, Germany

## Abstract

The present article reports the draft genome sequence of the strain *Komagataeibacter europaeus* CECT 8546, an acetic acid bacterium characterized by its ability to overproduce cellulose. This species is highly resistant to acetic acid and commonly found during vinegar elaboration.

## GENOME ANNOUNCEMENT

The acetic acid bacteria (AAB) are a group of Gram-negative bacteria that belong to the family *Acetobacteraceae*. They are obligate aerobic microorganisms that are able to produce acetic acid from ethanol and are the main bacteria responsible for vinegar production. The species *Komagataeibacter europaeus* (formerly *Gluconacetobacter europaeus* [1]) was first described in high-acetic acid vinegar in Germany and Switzerland (2). This species possesses a strong capacity to oxidize ethanol compared with other species of AAB (3), and it is associated with the production of vinegar in a submerged system (2, 4, 5).

The strain *K. europaeus* CECT 8546 was isolated from grape vinegar produced by the traditional method in the experimental cellar Mas dels Frares (Tarragona, Spain). Chromosomal DNA was isolated using the MasterPure complete DNA purification kit (Epicentre, Madison, WI, USA). For whole-genome sequencing, the Genome Analyzer II (Illumina, San Diego, CA, USA) and the 454 GS-FLX TitaniumXL (Titanium Chemistry, Roche Life Science, Mannheim, Germany) pyrosequencing systems were used. Preparation of shotgun libraries was performed according to the protocols of the manufacturers and resulted in 8,419,806 paired-end Illumina reads (112 bp) and 83,045 pyrosequencing reads. Initial hybrid *de novo* assembly using the MIRA software (6) resulted in 116 contigs (>500 bp) and an average coverage of 229-fold.

The genome of *K. europaeus* CECT 8546 consists of a chromosome with 4.11 Mb and an overall G+C content of 61.31%. Automatic gene prediction was performed using the software tool Prodigal (7). Genes coding for rRNA and tRNA were identified using RNAmmer (8) and tRNAscan (9), respectively. The Integrated Microbial Genomes-Expert Review (IMG-ER) system (10) was used for automatic annotation, which was subsequently manually curated using the Swiss-Prot, TrEMBL, and InterPro databases (11). The genome harbored 10 rRNA genes, 55 tRNA genes, 2,695 protein-coding genes with predicted functions, and 1,172 genes coding for hypothetical proteins. Among them, 51 genes encode dehydrogenases in different regions of the genome. It is noteworthy that one of these genes encodes for a NAD-dependent aldehyde dehydrogenase, which is a key enzyme during the conversion of ethanol to acetic acid (12). Moreover, a gene for a glucose dehydrogenase enzyme was also detected; this enzyme has been previously associated with vinegar flavoring (13).

In addition, the strain CECT 8546 presents the ability to biosynthesize cellulose very quickly. This biopolymer is the main component of the biofilm that AAB develop in the liquid-air interface during traditional vinegar elaboration (14). This biofilm keeps cells in tight contact with oxygen and provides a protective environment for them as they are subjected to extreme conditions (15). The gene that encodes the catalytic subunit required for cellulose biosynthesis is called cellulose synthase, and it is highly conserved among cellulose producer bacteria (16). In the genome of the strain CECT 8546, this gene is located in the cluster for starch and sucrose metabolism. The strain also presents an autoinducer synthase gene homologous to GinI and the regulator GinR (17).

### Nucleotide sequence accession numbers.

This whole-genome shotgun project has been deposited at DDBJ/EMBL/GenBank under the accession number LHUQ00000000. The version described in this paper is version LHUQ01000000.
